# How Much Can Diptera-Borne Viruses Persist over Unfavourable Seasons?

**DOI:** 10.1371/journal.pone.0074213

**Published:** 2013-09-04

**Authors:** Maud V. P. Charron, Thomas Balenghien, Henri Seegers, Michel Langlais, Pauline Ezanno

**Affiliations:** 1 UMR1300 Biologie, Epidémiologie et Analyse de Risques en santé animale, INRA, LUNAM Université, Oniris, Ecole nationale vétérinaire, agroalimentaire et de l’alimentation Nantes-Atlantique, Nantes, France; 2 UMR 5251, Université de Bordeaux, IMB, Bordeaux, France; 3 UMR 5251, CNRS, IMB, Talence, France; 4 UMR1309 CMAEE, INRA, CIRAD, Montpellier, France; Ecole des Mines d’Alès, France

## Abstract

Diptera are vectors of major human and animal pathogens worldwide, such as dengue, West-Nile or bluetongue viruses. In seasonal environments, vector-borne disease occurrence varies with the seasonal variations of vector abundance. We aimed at understanding how diptera-borne viruses can persist for years under seasonal climates while vectors overwinter, which should stop pathogen transmission during winter. Modeling is a relevant integrative approach for investigating the large panel of persistence mechanisms evidenced through experimental and observational studies on specific biological systems. Inter-seasonal persistence of virus may occur in hosts due to viremia duration, chronic infection, or vertical transmission, in vector resistance stages, and due to a low continuous transmission in winter. Using a generic stochastic modeling framework, we determine the parameter ranges under which virus persistence could occur via these different mechanisms. The parameter ranges vary according to the host demographic regime: for a high host population turnover, persistence increases with the mechanism parameter, whereas for a low turnover, persistence is maximal for an optimal range of parameter. Persistence in hosts due to long viremia duration in a few hosts or due to vertical transmission is an effective strategy for the virus to overwinter. Unexpectedly, a low continuous transmission during winter does not give rise to certain persistence, persistence barely occurring for a low turnover of the susceptible population. We propose a generic framework adaptable to most diptera-borne diseases. This framework allows ones to assess the plausibility of each persistence mechanism in real epidemiological situations and to compare the range of parameter values theoretically allowing persistence with the range of values determined experimentally.

## Introduction

The unfolding of the seasons generates profound, cyclical environmental changes, presenting organisms with multiple challenges which are the most difficult and diverse that they must face [1]. Adaptations to seasonal changes – reproduction, migration, and dormancy periods – are a fundamental feature shared by all living organisms. Insects, which are particularly sensitive to their environment and are present all over the world, encounter extremely varied climate conditions. The adaptative “strategies” they have developed and their physiological consequences reveal a formidable diversity between species, and even between populations of the same species living at different latitudes [2]. Insects must in particular survive unfavourable seasons, such as the winter in temperate climates and the hot dry season in tropical climates [3]. During this unfavourable period, many enter diapause. This is a state regulated by the endocrine system characterized by low metabolic activity associated with a reduction of morphogenesis, an increase in resistance to extreme conditions, and a modification of activity (dormancy or migration). Diapause most often occurs at a genetically determined point in the lifecycle in response to environmental signals announcing unfavourable conditions [1].

Hematophagous insects, which have the distinction of being vectors of pathogens that cause disastrous diseases for human and animal health (malaria, dengue, African horse sickness, etc.), are no exception to this rule. One of the most striking features of vector-borne diseases is their strong seasonality connected to that of vectors, leading to a quasi-disappearance of clinical cases during the unfavourable season. The pathogens transmitted therefore must develop a persistence strategy to survive the unfavourable season and to adapt to their vectors’ own seasonal dynamics. Conceptually, three independent mechanisms are possible: *i)* low continuous transmission associated with the survival and residual biting activity of the adult vector, *ii)* persistence in the host, and *iii)* persistence in the resistance stages of the vector. Continuous transmission could occur in areas where vectors bite hosts year round; with low winter temperatures lengthening the interval between two meals and the extrinsic incubation period (latency period), clinical cases are rare and may pass unnoticed. This mechanism was suggested to constitute a means of persistence in the *Culex*/West Nile virus system in southern California [4]. Persistence in a host may be related to long viremia, to vertical transmission, or to a chronic infection phenomenon with resurgent viremia. For example, in the *Culicoides*/bluetongue virus system, cattle present a long viremia and the transmission of the virus from a cow to her calf is possible [5], [6]. In the *Culex*/Western equine encephalitis virus system, a resurgence of viremia was observed experimentally in snakes after hibernation [7]. A pathogen also can persist if it maintains itself in the overwintering stages of the vector after vertical transmission, in the eggs or in newly emerged adults, as is the case respectively of the mosquito/Japanese encephalitis virus system and the mosquito/St. Louis encephalitis virus system [8], [9].

The existence of each of these mechanisms has been demonstrated, experimentally and by field observations, in different biological systems. In contrast, few studies have investigated several of these pathogen persistence mechanisms, illustrating the difficulty of demonstrating the efficacy of a mechanism and of quantifying its importance under natural conditions [4]. Little progress thus has been made to contradict Léon Rosen, who wrote in 1987: “at present, the mechanism by which mosquito-borne alphaviruses pass the winter is obscure” [10]. Yet the persistence of arboviruses represents an interesting model of a parasite (in the broad sense) developing adaptation mechanisms to the seasonal synchronization of host/vector pairs (with the latter also possible to consider as a parasite). Modelling approach allows us to better understand the reasons for the selection of a given overwintering mechanism and may help us to devise experiments to quantify critical parameters of these overwintering mechanisms. Furthermore, the understanding of these mechanisms has direct consequences in terms of the prevention of vector-borne diseases (vaccination strategies, etc.), which strongly impact the health of human and animal populations.

Our objective was to assess parameter ranges under which persistence could occur in several virus/vector/vertebrate systems and then to compare the range of parameter values theoretically allowing persistence with the range of values determined experimentally.

## Materials and Methods

### The General Model

**Figure 1:**
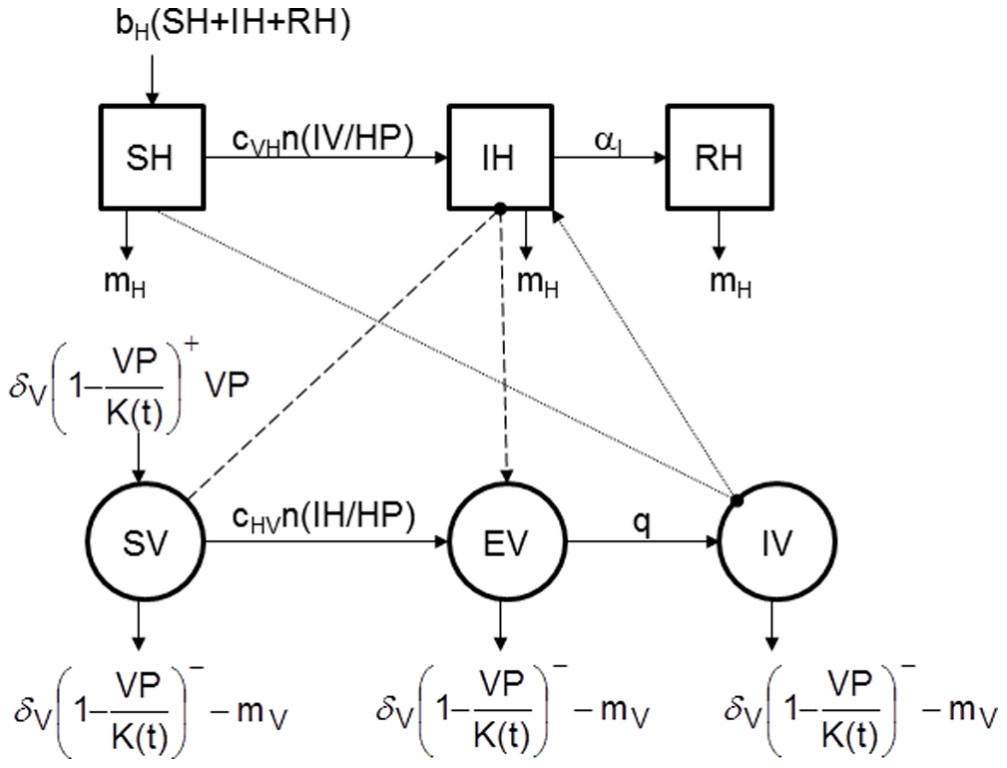
Conceptual model of the spread of a vector-borne pathogen during favourable period. Squares represent the health states of hosts, circle those of vectors. Solid lines represent transitions between health statuses, dashed lines represent the host-to-vector transmission (when a susceptible vector (*SV*) bites an infectious host (*IH*) and becomes exposed but not yet infectious *(EV)*), and dotted lines represent the vector-to-host transmission (when an infectious vector (*IV*) bites a susceptible host (*SH*) which becomes infectious (*IH*)). Parameters are defined in Table 1.

To study these different mechanisms we developed a general common model for all of the mechanisms of virus persistence. Modifications due to each hypothesis are described thereafter. We used a standard compartment model to describe the vector-borne transmission of a pathogen between a vertebrate host population (*HP*) and a vector population (*VP*) (Figure 1). The parameters of this model are defined in Table 1. In the presence of a virus, the host population (*HP*) is divided into three health states (Figure 1): susceptible (*SH*), infectious (*IH*), and immune (*RH*). It is assumed to remain constant: the entry rate (*b_H_*) compensates the exit rate (*m_H_*). For our study to be relevant to a wide range of vector-borne diseases, three different demographic regimes of hosts were represented: one with a relatively high turnover adapted to a bird population, a second with an average turnover comparable to that of a ruminant livestock population such as cattle, and a third with a low turnover comparable to a human population.

**Table 1:** Model parameter values.

Host parameters	Description	Value			
*b_H_*	Turnover rate (days)	Humans = 1/(70×365)			
		Cattle = 1/(5×365)			
		Birds = 1/(2×365)			
*1/α_I_*	Viremia duration (days)	6			
*c_VH_*	Vector/host transmission probability	0.8			
**Vector parameters**	**Description**	**Value**			
*m_V_*	Mortality rate (1/day)	1/21			
*δ_V_*	Density-dependant growth rate	1			
*K(t)*	Carrying capacity	*K(t) = *1_[1;d]_(*t*)×[*h*×sin(|π(365-*t*)/*d*|)+*Nb* ] +1_[d+1;365]_(*t*)× *Nb*			
*h*	Maximum of *K(t)*	10^9^			
*d*	Duration of favourable period (days)	243			
*Nb*	Number of vectors emerging after the unfavourable period	10^5^			
*Nb_unf_*	Number of vectors present at the beginning of the unfavourable period	10^5^			
*c_HV_*	Vector/host transmission probability	0.5			
*n*	Biting rate in the favourable period	1/7			
*1/q*	Duration of extrinsic incubation period in the favourable period (days)	10			
**Persistence mechanism parameters**	**Description**	**Value**			
		**Min**	**Max**	**No. values**	**No. scenarios**
*x*	Maximum viremia duration	123	160	38	38*3*5 =
*M*	Duration of most probable viremia duration	2	10	3	570
*A*	Tail of the distribution area	0.01	0.03	5	
*f_I_*	Probability of chronic infection	0	1	50	50*7*6 =
*p*	Rate of occurrence of a return to a viremic state	0	1	7	2100
*1/α_IC_*	Duration of chronic infection (days)	30	730	6	
*g_H_*	Probability of vertical transmission in the host	0	1	47	47
*g_V_*	Probability of transovarian transmission in the vector	0	1	29	29*5*5 = 725
*inter*	Length of the period during which the number of infected vectors entering diapause is calculated	1	243	5	
*Nb_d_*	Number of vectors experiencing diapause (no mortality)	10^3^	10^5^	5	
*n_unf_*	Biting rate in the unfavourable period	1/100	1/7	6	6*6*17 =
*1/q_unf_*	Duration of extrinsic incubation period in the unfavourable period (days)	10	100	6	612
*m_Vunf_*	Mortality rate during the unfavourable period (days)	1/100	1/21	17	

In the presence of a virus, the vector population (*VP*) is divided into three health states (Figure 1): susceptible (*SV*), exposed (*EV*), and infectious (*IV*). The vectors have a mean lifespan of 1/*m_V_*. In a disease-free environment, the density-dependant growth of this population is regulated by the seasonal carrying capacity *K(t)*. If *VP*<*K(t)*, susceptible vectors are born, if *VP*≥*K(t)*, there is excess mortality of vectors in each health state. In a period favourable for vectors, the *K* function is a sinusoidal function with a maximum *h*. In an unfavourable period, the *K* function is assumed constant and equal to *Nb*. These *Nb* vectors are not taken into account in the virus transmission dynamic in this general model, but represent the number of new vectors emerging when the favourable period returns. The vector-borne transmission takes place when an infectious vector (*IV*) bites a susceptible host (*SH*) which then becomes infectious (*IH*), or when a susceptible vector (*SV*) bites an infectious host (*IH*) and then becomes exposed but not yet infectious *(EV)*. The dynamics of hosts and vectors are described by the following ODE system (Eq. (1)):
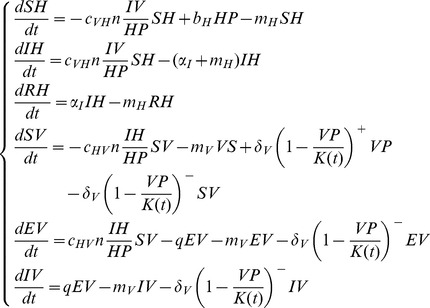
(1)


Very close models have been used to represent a large panel of vector-borne diseases, such as dengue, West Nile fever and bluetongue [11], [12], [13].

The stochastic version of the ODE model was developed to enable us to study persistence phenomena, based on random events. Each transition rate of the ODE (*e.g. α_I_*) was transformed into a transition probability (*p_αI_* = *1-e^−αIΔt^*, with *Δt = *1day). Then, the number of transitions between two given health statuses at time *t* was represented by a random variable and followed a Binomial distribution, which parameters were the population size in the status concerned and the associated transition probability. For example, for two theoretical health statuses (*X*, *Y*) and associated transition probability *p*(*X* *Y*), we would have:
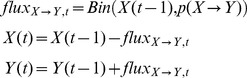
(2)


Only the equation regulating vector population birth follows a Poisson probability distribution with parameter 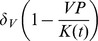.

### Outputs

The probability of virus persistence 5 years after virus introduction (named hereafter persistence) and the distribution of the extinction dates allowed us to describe the virus capacity to persist over unfavourable periods. Persistence is estimated by the proportion of repetitions in which the virus is still present (either in the host or in the vector populations) 5 years after its introduction. The extinction dates for each repetition correspond to the date when the virus has disappeared from both populations (hosts and vectors). These dates allow the calculation of the proportion of repetitions in which the virus has disappeared per year since the virus introduction. Simulations were carried out over 5 years and not less to study the persistence of the virus and not its invasion capacity.

### The different Persistence Mechanisms

To explore each persistence mechanism, we modified the general model by successively changing one or several features. For each mechanism, we studied the virus persistence and the distribution of extinction dates 5 years after its introduction. We assessed parameter ranges under which persistence could occur according to the host demographic regime.

#### Long host viremia

The duration of viremia, meaning the period during which the virus is present in the blood, has been relatively well documented, although the period during which viremia is sufficient to infect vectors is understood less well. Viremia can be extremely variable between individuals in the same population; for example, the viremia of bluetongue virus infection in cattle ranges from 7 to 63 days [14], and longer viremias have been described on an exceptional basis [15]. Of course, persistence seems possible only if viremia duration can exceed the duration of the unfavourable period. However, it is not known to what extent persistence is probable over several years according to the proportion of individuals having a long viremia duration, or simply to the maximum of the viremia duration.

**Figure 2:**
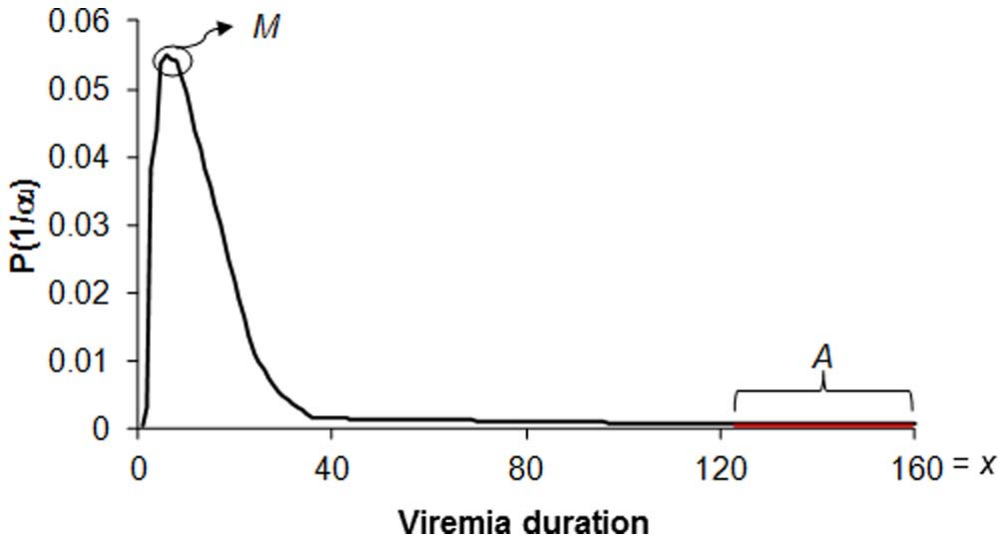
Distribution function depending on the different probabilities of viremia durations. *M* = 6 days, *A* = 0.03 and *x* = 160 days.

To represent this variability, we attributed a viremia duration to each newly infected individual, using a discrete random variable with an asymetric unimodal distribution. Therefore, we designed qualitatively a parametric function. The definition of this function is [1; *x*] (Table 1), *x* being greater than the duration of the unfavourable period (365-*d*) (Figure 2). Two other parameters manage this function (Table 1): the most likely viremia duration, *M* (*i.e.* the distribution function mode), which is generally short, and the cumulative probability to have a viremia duration longer than (365-*d*), *A* (Figure 2).

On the interval of tested values (Table 1), we assessed 570 scenarios for each host population, or, in other words, for each of the three demographic regimes of hosts (1710 scenarios in total).

#### Reactivation of viremia during persistent infection in hosts

It has long been known that certain arboviruses can be rediscovered in a host weeks or even months after infection, even in the case of brief viremia [16]. Under certain systems, a resurgence of viremia was observed in these infected hosts [7]. Of course, persistence seems possible if a chronically infected individual become viraemic again while the virus is not present any more. However, it is not known to what extent persistence is probable over several years according to the probability of chronic infection, or to the duration chronic infection.

**Figure 3:**
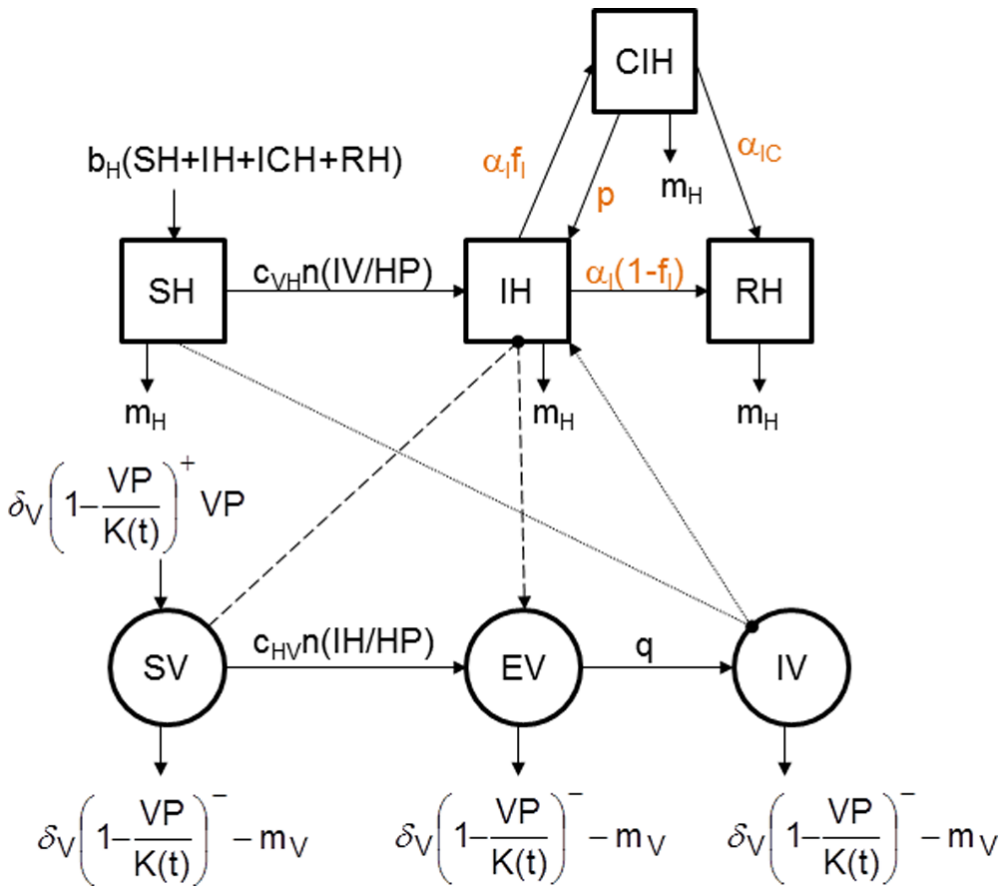
Conceptual model of the spread of a vector-borne pathogen during favourable period with reactivation of viremia during persistent infection in hosts. Squares represent the health states of hosts, circle those of vectors. Solid lines represent transitions between health statuses, dashed lines represent the host-to-vector transmission (when a susceptible vector (*SV*) bites an infectious host (*IH*) and becomes exposed but not yet infectious *(EV)*), and dotted lines represent the vector-to-host transmission (when an infectious vector (*IV*) bites a susceptible host (*SH*) which becomes infectious (*IH*)). In orange, modifications in model due to the mechanism compared with the conceptual model in favourable period without any mechanism (Figure 1).

To represent this mechanism, a fourth host health state (Figure 3), chronically infected individuals (*CIH*), was added to the shared model and the equations for hosts (Eq.1) were modified as follows:
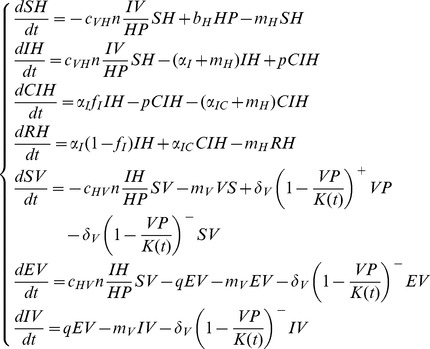
(3)


In this way, infectious hosts (*IH*), once their viremia ends at rate *α_I_*, either will be chronically infected (*CIH*) with probability *f_I_*, or will be cured (*RH*) *(1-f_I_)*. Following stress (period of reproduction, migration, other diseases), chronically infected hosts (*CIH*) can become viraemic again (*IH*), with occurrence rate *p*. Independently, they exit the *CIH* compartment at rate *α_IC_*. In the stochastic version of the model, these transitions follow binomial probability distributions.

On the interval of tested values (Table 1), we assessed 2100 scenarios for each of the three demographic regimes of hosts (6300 scenarios in total).

### Vertical Transmission in Hosts

In the three host regimes considered, only those corresponding to humans and cattle, with 9 month gestation periods, can allow virus persistence over the period unfavourable for vectors through vertical transmission, and therefore the birth of a viraemic individual. In contrast, the demographic regime corresponding to a bird population is not taken into account in this mechanism as the incubation time and egg laying periods in temperate environments do not allow to pass the unfavourable season.

Of course, persistence seems possible if a viraemic individual is born while the virus is not present anymore. However, it is not known to what extent persistence is probable over several years according to the probability of vertical transmission.

Infectious hosts (*IH*) are divided into two categories according to whether vertical transmission is possible (with a probability *g_H_*) or not, vertical transmission only being possible during gestation (probability *b_H_*). Infectious individuals for which there is vertical transmission are distributed at infection uniformly over the gestation stages. In the stochastic version of the model, these transitions follow binomial probability distributions.

On the interval of tested values (Table 1), we assessed 47 scenarios for each of the two demographic regimes (cattle and human; 94 scenarios in total).

#### Transovarian transmission in vectors prior to diapause

Diapause may take place in vector diptera during the egg, larval, or adult stage depending on the genus and species considered [3]. Two main mechanisms may be distinguished. In the first, overwintering takes the form of quiescent eggs, as is the case for most *Aedes* genus mosquitoes [17], where infectious vectors present over the entire favourable period lay potentially infected eggs which will be able to contribute to the emergence of infectious vectors in the next favourable period. In the second, the overwintering forms are adults (or larvae), as is the case for most *Culex* genus mosquitoes [17], among which the overwintering females are nulliparous, inseminated and with inhibited trophic behaviour [18], [19]. Only vectors present at the end of the favourable period give birth to potentially infected adults which will contribute to the emergence of infectious vectors in the next favourable period.

Of course, persistence seems possible if infected vectors emerge in the next favourable period. However, it is not known to what extent persistence is probable over several years according to the period during which the transovarian transmission is present, or to its probability.

These mechanisms are driven by the length of the favourable period during which the vertical transmission is present (*inter*), the probability of transovarian transmission (*g_V_*) and the total number of individuals entering diapause (*Nb_d_*). These parameters determine the share of infectious vectors whose offspring will be infectious, and therefore the proportion of infectious vectors among those emerging in the following favourable period. In the stochastic version of the model, these transitions follow binomial probability distributions.

On the interval of tested values (Table 1), we assessed 725 scenarios for each of the three demographic regimes of hosts (2175 scenarios in total).

#### Low continuous transmission in the unfavourable period

This mechanism involves a low vector-borne transmission during the unfavourable period. This could take place when insects maintain a residual biting activity or when a gonotrophic discordance phenomenon, when blood meals are maintained but there is no ovarian maturation, is observed, as may be the case among the *Anopheles* genus mosquitoes in temperate regions [20].

Of course, persistence seems possible if vectors maintain a biting activity during the unfavourable period. However, it is not known to what extent persistence is probable over several years according to the number of present vectors and to their gonotrophic parameters.

**Figure 4:**
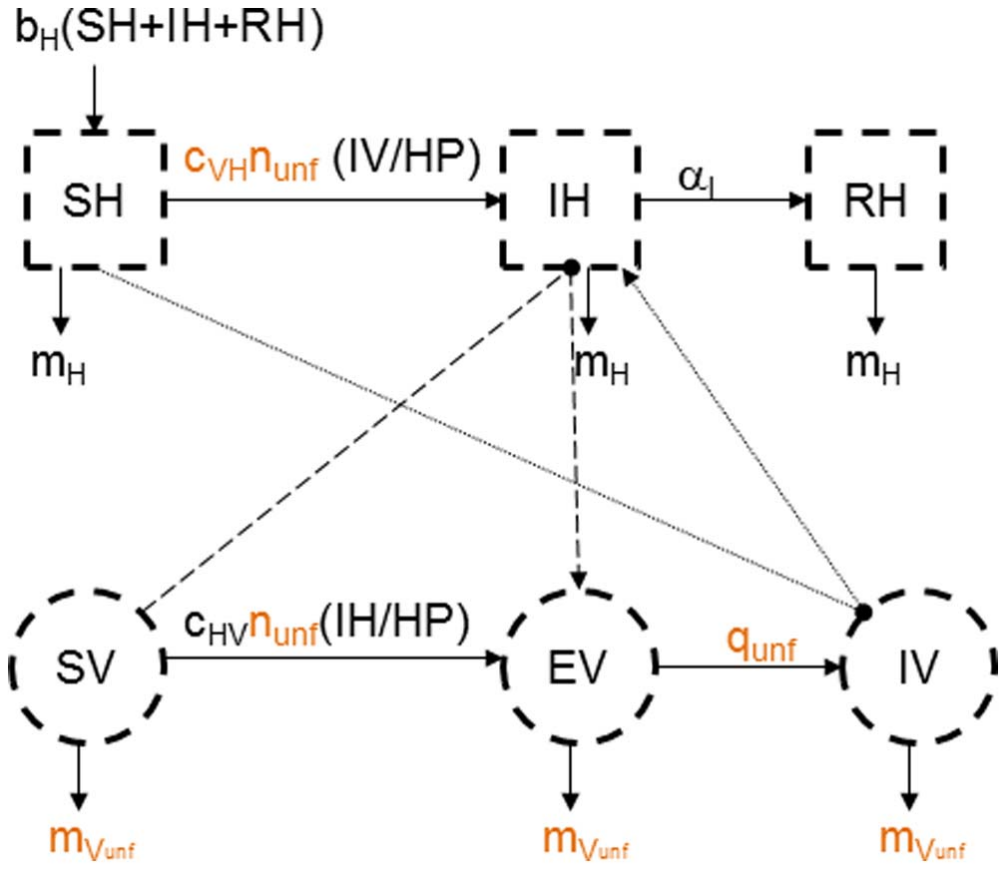
Conceptual model of the spread of a vector-borne pathogen during unfavourable period with a low continuous transmission. Dotted squares represent the health states of hosts, dotted circle those of vectors. Solid lines represent transitions between health statuses, dashed lines represent the host-to-vector transmission (when a susceptible vector (*SV*) bites an infectious host (*IH*) and becomes exposed but not yet infectious *(EV)*), and dotted lines represent the vector-to-host transmission (when an infectious vector (*IV*) bites a susceptible host (*SH*) which becomes infectious (*IH*)). In orange, modifications due to a low continuous transmission compared with the conceptual model in favourable period without any mechanism (Figure 1).

**Figure 5:**
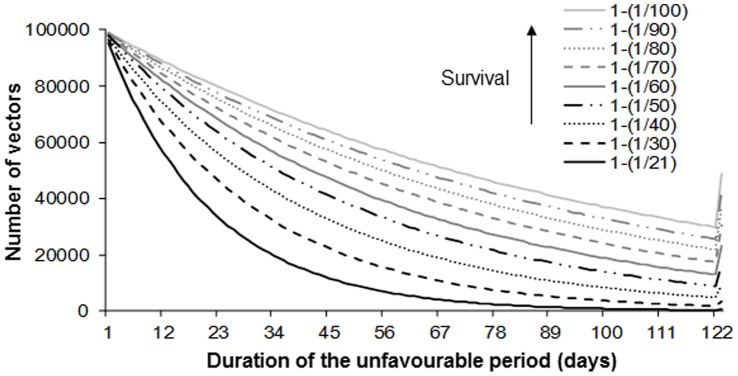
Variation of vector number during the unfavourable period depending on their survival rate (1-*m_Vunf_*).

The function *K(t)* managing the seasonality is unchanged but the vectors present in constant numbers (*Nb*) during the unfavourable period now are involved in the virus spread dynamics (*Nb_unf_*). These *Nb_unf_* vectors are distributed in the different health states in proportions equal to those at the end of the favourable period (Figure 4). However, the unfavourable period is marked by a slowdown in their pace of activity and a complete absence of egg laying. In consequence, while their survival rate (*1-m_Vunf_* >1-*m_V_*, Table 1 and Figure 5) and their extrinsic incubation period (*1/q_unf_* >*1/q*, Table 1) increase, their biting rate diminishes (*n_unf_* <*n*, Table 1). In the stochastic version of the model, these transitions follow binomial probability distributions.

On the range of values tested (Table 1), we assessed 612 scenarios for each of the three demographic regimes of hosts (1836 scenarios in total).

### Initial Conditions

The initial conditions were set to achieve over a favourable period a mean percentage of 5% infectious hosts and a mean percentage of 5‰ infectious vectors. The initial numbers are *SH_0_* = 10^8^, *IH_0_* = 1 and *SV_0_* = 10^5^, the other state variables being null. The transmission parameter values were determined to be compatible with numerous vector-borne diseases [12], [14], [21]–[27]. An initial sensitivity analysis made it possible to verify that in the absence of one of the various persistence mechanisms considered, the virus could not survive the first period unfavourable for its vector, even with extreme transmission parameter values (not shown).

Furthermore, in order to eliminate early extinctions resulting from the stochasticity of the model, we only kept the runs that did not extinguish before the last day of the first favourable period. For each scenario of each hypothesis, 50 repetitions were retained and the time step is the day.

## Results

### Long Host Viremia

**Figure 6:**
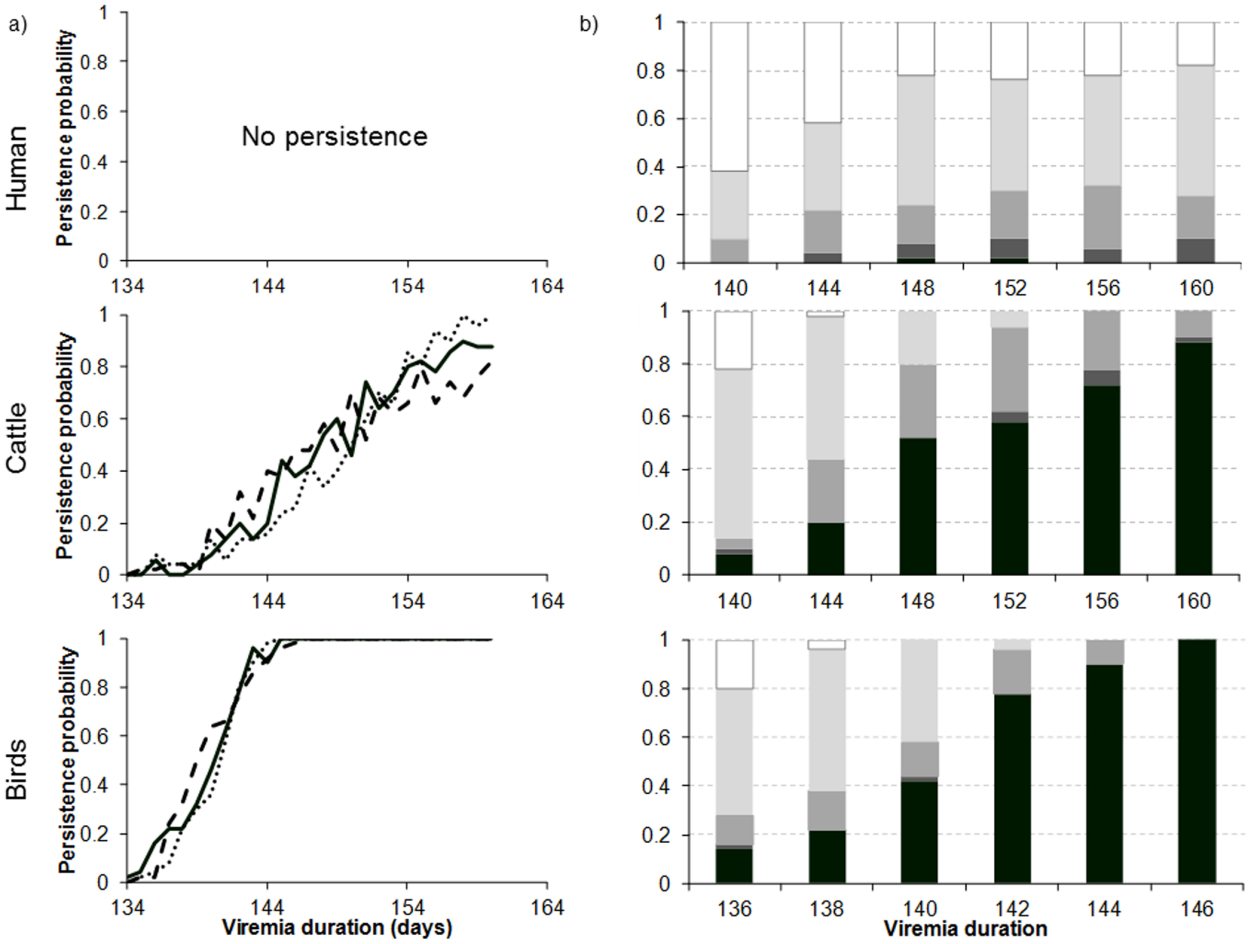
Persistence (a) and distribution of extinction dates (b) as a function of host regime, maximum duration (*x*) and most likely duration (*M*) of the viremia. a) solid line, *M* = 6 days; dashed line *M* = 2 days; dotted line *M* = 10 days. *A* = 0.01. b) *M* = 6 days and *A* = 0.01. In black, persistence. In shades of grey, from darkest to lightest, extinction the 5^th^, 4^th^, 3^rd^ year after introduction, respectively. In white, extinction the 2^nd^ year.

Persistence increases with the maximum duration of viremia (*x*), with a higher increase for a faster renewal of the host population (Figure 6a). When the virus confers a long-lasting immunity and the population turnover rate is low (case of humans), the virus cannot persist over 5 years (Figure 6a). Hence, for a maximum viremia duration greater than 144 days, the persistence periods are stable (Figure 6b). As nearly the entire population becomes resistant, transmission is rendered impossible. For the other demographic regimes, the mode of the distribution function (Figure 6a) and the cumulated probability of long viremia do not change the duration of persistence (data not shown). In contrast, persistence gradually increases with the maximum duration of viremia, with a higher increase for a renewal of the host population. With a maximum viremia duration of 148 days in 1% of infectious individuals, the persistence of the virus after 5 years is certain in birds and in half the cases in cattle (Figure 6).

### Reactivation of Viremia during Persistent Infection in Hosts

**Figure 7:**
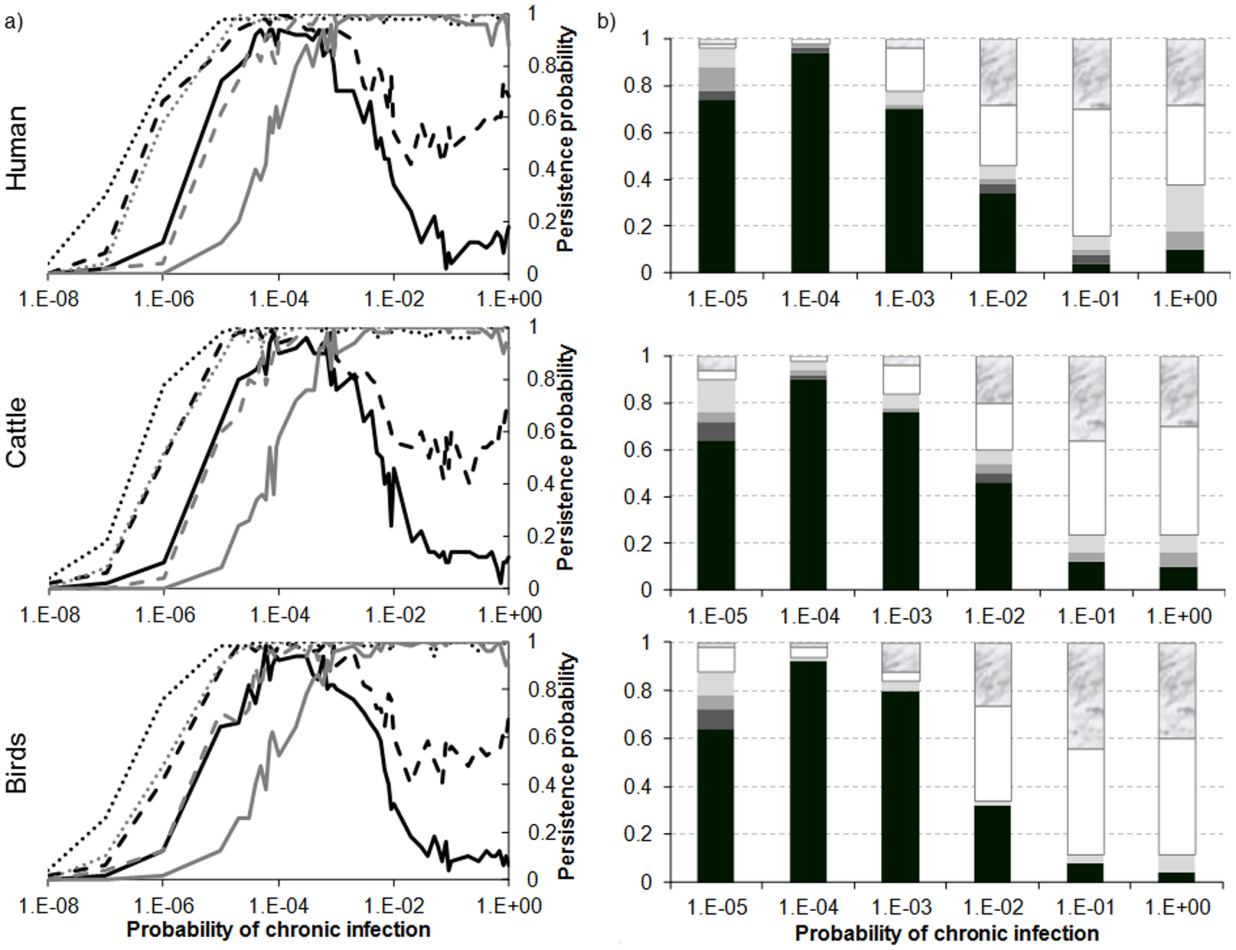
Persistance (a) and distribution of extinction dates (b) as a function of host regime, probability of chronic infection (*f_I_*), duration of chronic infection (1/*a_IC_*) and probability of a return to the viraemic stage (*p*). a) in black *1/α_IC_* = 1 year, in grey *1/α_IC_* = 1 month: solid line p = 10*^−^* ^4^; dashed line p = 10*^−^* ^3^; dotted line p = 10*^−^* ^2^. b) *1/α_IC_* = 1 year and *p* = 10*^−^* ^4^. In black, persistence. In shades of grey, from darkest to lightest, extinction the 5th, 4th, 3rd year after introduction, respectively. In white, extinction the 2nd year. In marble, extinction the 1^st^ year.

Regardless of the host demographic regime, persistence increases with the probability of chronic infection (*f_I_*) up to a threshold of 10*^−^* ^3^ at which it reaches certainty. After this point, persistence decreases if the duration of chronic infection is long (1/*α_IC_* >3 months) and the probability of a return to a viraemic stage is low (*p*<10*^−^* ^2^) (Figure 7a). Hence, there is an interaction between these three parameters. Numerous scenarios with a wide range of parameters were tested. The distribution of extinction dates (Figure 7b) confirms this threshold (*f_I_* = 10*^−^* ^3^) with an increase of early extinction dates after this value.

### Vertical Transmission in Hosts

**Figure 8:**
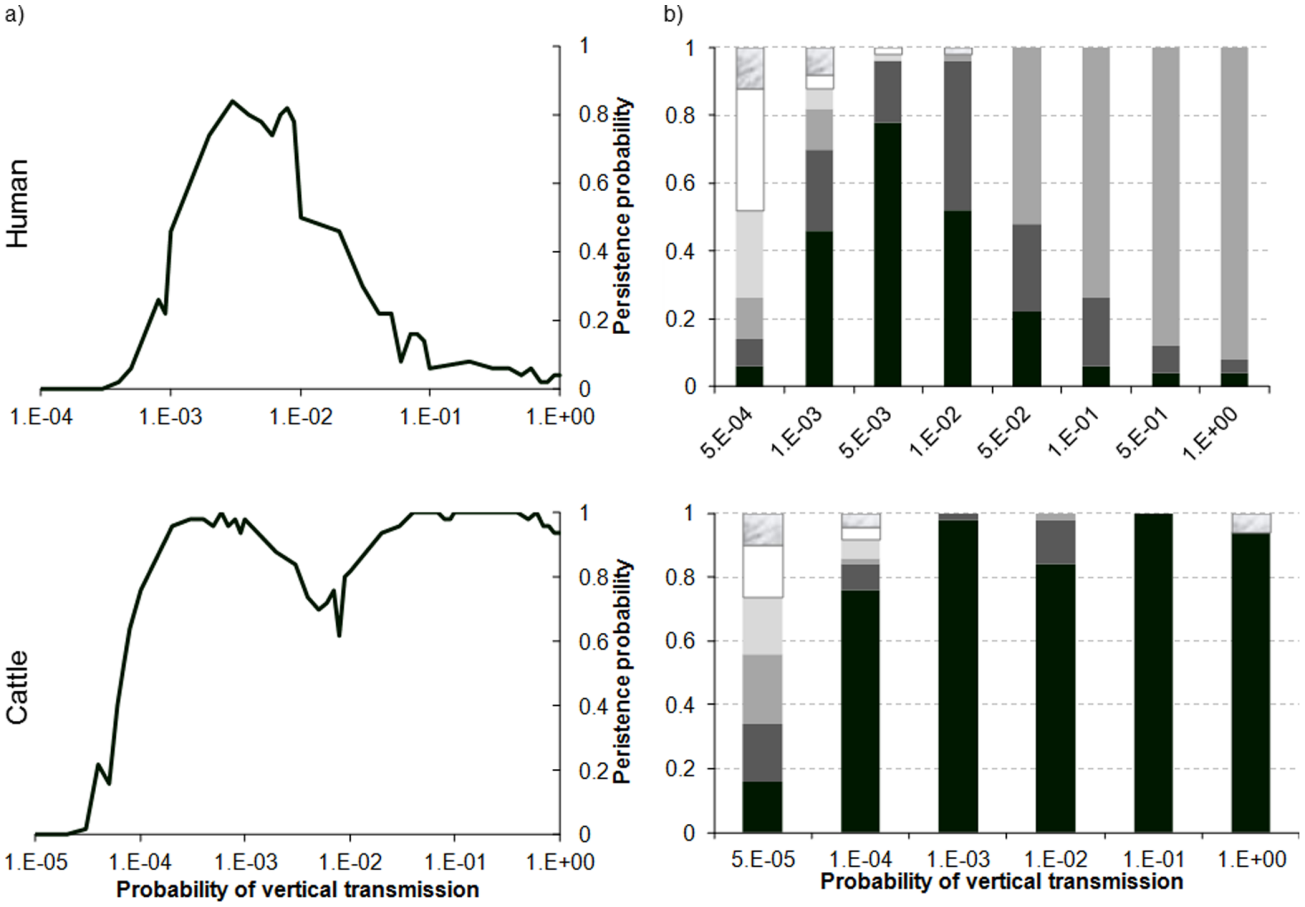
Persistence (a) and distribution of extinction dates (b) as a function of host regime and probability of vertical transmission in the host (*g_H_*). b) in black, persistence. In shades of grey, from darkest to lightest, extinction the 5th, 4th, 3rd year after introduction, respectively. In white, extinction the 2nd year. In marble, extinction the 1st year.

Persistence varies non-linearly with the probability of vertical transmission (*g_H_*), the relation depending on the host demographic regime (Figure 8a). For humans, persistence increases with the probability of vertical transmission (*g_H_*) up to the threshold *g_H = _*10*^−^* ^2^, progressively diminishing thereafter. For cattle, persistence increases with *g_H_*. It is constant and equal to 1 as soon as *g_H_* >0.5×10*^−^* ^3^, with the exception of *g_H_* values between 10*^−^* ^3^ and 10*^−^* ^2^ (Figure 8a). However, the dates of extinction show that in all cases the virus only disappears late in the two populations (Figure 8b).

### Transovarian Transmission in Vectors Prior to Diapause

**Figure 9:**
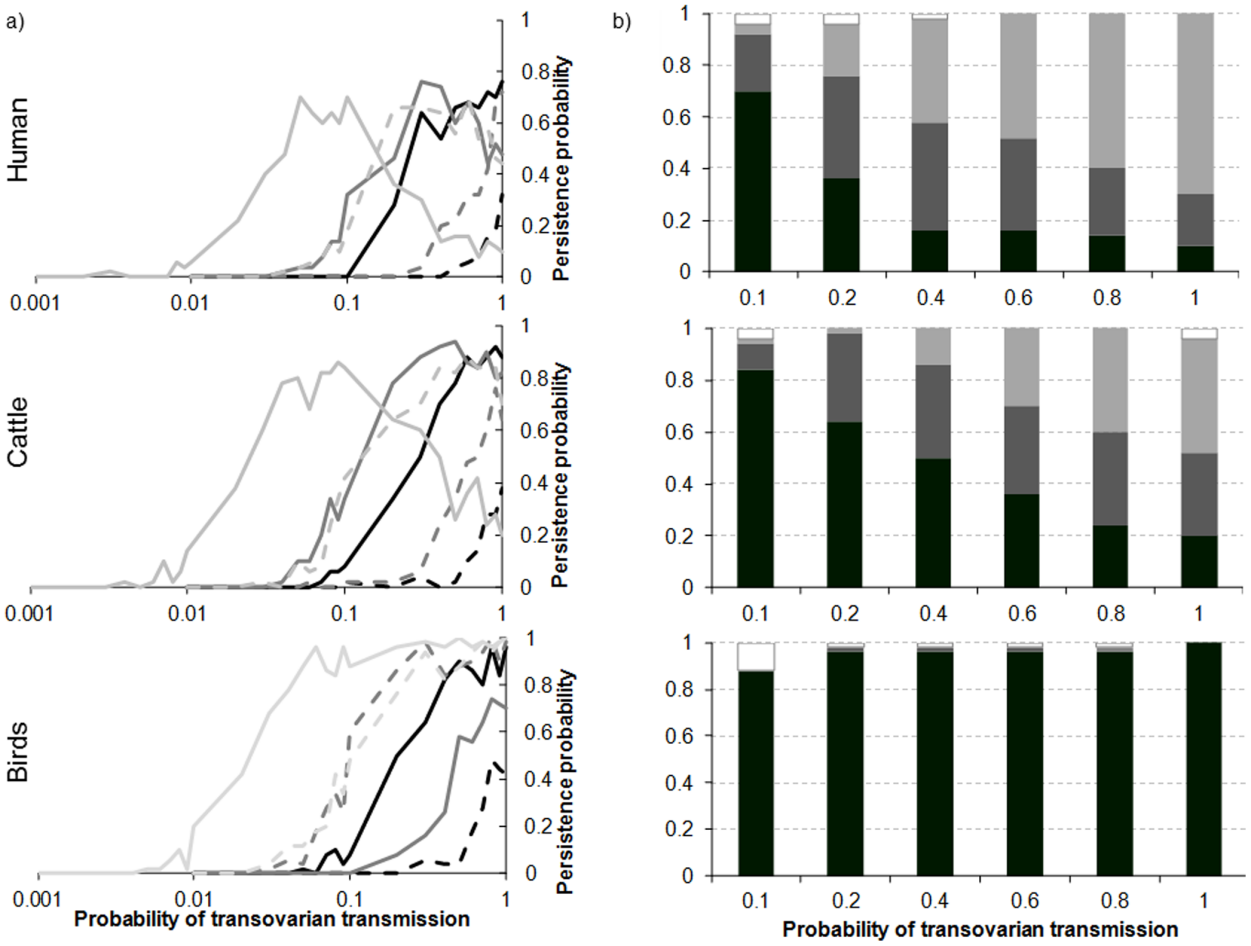
Persistence (a) and extinction dates (b) as a function of host regime, probability of transovarian transmission in the vector (*g_V_*), length of the period on which is calculated the population of infected vectors entering diapause (*inter*), and number of vectors emerging after the unfavourable period (*Nb_d_*). a) in black *inter* = [1: 243], in dark grey *inter* = [123: 243], in light grey *inter* = [230: 243]: solid line *Nb_d_* = 50000; dashed line *Nb_d_* = 10000. b) *Nb_d_* = 50000 and *inter* = [230: 243]. In black, persistence. In shades of grey, from darkest to lightest, extinction the 5th, 4th, 3rd year after introduction, respectively. In white, extinction the 2nd year.

Persistence varies non-linearly with the probability of transovarian transmission (*g_V_*), the number of vectors (*Nb_d_*) and the length of the period over which the population of infected vectors entering diapause (*inter*) is calculated, the relation depending on the host demographic regime (Figure 9a). When the proportion of diapausing infected vectors was calculated with vector population present in the whole favourable period (*inter* = [1: 243]), persistence doubles when *Nb_d_* is multiplied by 5, (Figure 9a) for the three host regimes considered. When this proportion was calculated with vectors present at the end of the favourable period only (*inter* = [230: 243]), persistence decreases as soon as *g_V_* >0.1 for cattle and humans if *Nb_d_ = *50 000 (Figure 9a). Hence, there is an interaction between these three parameters. However, despite the decrease in persistence, the dates of extinction show that the virus persists up to the 4^th^ or 5^th^ year after its introduction (Figure 9b).

### Low Continuous Transmission in the Unfavourable Period

**Figure 10:**
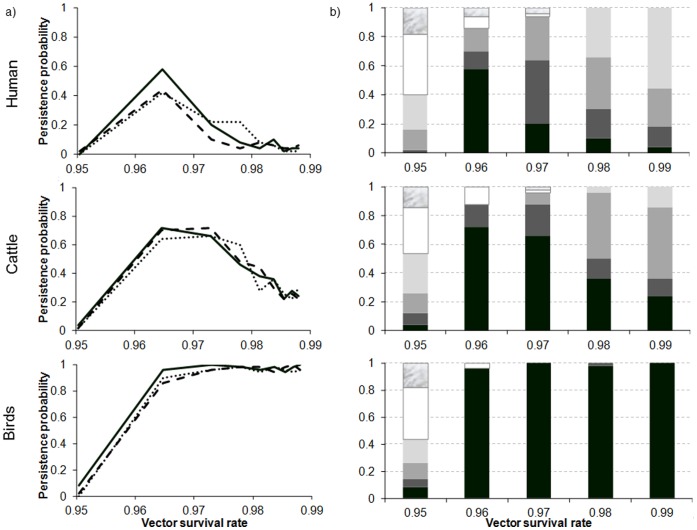
Persistence (a) and the distribution of extinction dates (b) as a function of host regime, and survival rate of the vector (*1-m_V_*), duration of extrinsic incubation period (*1/q_unf_*), and biting rate (*n_unf_*) during the unfavourable period. a) solid line, most likely values 1/*q_unf_* = 20 days and *n_unf_* = 1/20; dashed line same values as in the favourable period 1/*q_unf_* = 10, *n_unf_ = *1/7; dotted black line extreme values 1/*q_unf_* = 100, *n_unf_* = 1/100. b) 1/*q_unf_* = 20, *n_unf_ = *1/20. In black persistence. In shades of grey, from darkest to lightest, extinction the 5th, 4th, 3rd year after introduction, respectively. In white, extinction the 2nd year. In marble, extinction the 1st year.

Persistence varies non-linearly with the survival of the vector in winter (1-*m_Vunf_*), the relation depending on the host demographic regime (Figure 10a). In contrast, variations in the biting rate (*n_unf_*) and in the duration of the extrinsic incubation period (*1/q_unf_*) do not modify persistence.

In a human population, virus persistence increases with the winter survival of the vector (Figure 10a), to achieve 60% for a vector winter survival of 30 days, then diminishes. In a cattle population, virus persistence follows the same profile up to 70% for a vector winter survival of between 30 (1-*m_Vunf_* >0.967) and 40 days (1-*m_Vunf_* >0.975), then diminishes for higher survival durations (Figure 10a). In a bird population, a vector winter survival longer than one month leads to certain persistence of the virus (Figure 10a). Despite the reduction in persistence in human and cattle populations, the dates of extinction are late and show virus persistence beyond three years as soon as the vector has a lifespan longer than one month (Figure 10b).

## Discussion

A modelling approach allowed us to assess the likelihood of mechanisms allowing an arbovirus to persist beyond the unfavourable period for its vector according to the parameters driving these mechanisms and the host demographic regime. Experimental studies and field observations indeed render it possible to demonstrate the existence of a persistence mechanism and to quantify the parameters of this mechanism [4], [9]. Review articles offer lists of hypotheses for the persistence of a disease virus without exploring in detail their likelihood [10], [28]. In contrast, modelling is a relevant approach for investigating an ensemble of persistence mechanisms and to compare the range of parameter values allowing a theoretical persistence with the range of values determined experimentally.

We evaluated 5 mechanisms of virus persistence related either to the persistence in the host, to the persistence in the vector, or to a low continuous transmission, and we identified the parameter values that theoretically allow virus persistence over several years. For each of the mechanisms, two types of threshold effects were observed. The first leads to a certain persistence, for the highest parameter values and/or for a demographic regime with the highest turnover, or, in other words, that comparable to a bird population. The second leads to a reduction in persistence beyond a certain value of the parameters driving the mechanism. This threshold effect is influence by the host demographic regime: if this proves to be insufficient and does not renew the susceptible population sufficiently quickly, persistence diminishes. Only a demographic regime with a high turnover comparable to that of birds allows the permanent renewal of the susceptible population. Such hosts often show strongly seasonal reproduction pattern. This seasonality may limit however contacts between susceptible hosts and vectors and thus reduce the probability of persistence.

The persistence mechanism of the virus in the host can ensure, for certain parameter values, virus persistence. In the cattle/bluetongue virus system, long viremia, lasting 7 to 63 days and exceptionally more, could be observed [14], [15]. As these data mainly were produced by experimental infections involving a limited number of animals (from a few to about twenty), Singer *et al.* [14] estimated that the probability of a viremia longer than 70 days was between 0 and 0.05 depending on the modes of calculation. Yet we observed a theoretical 5 year virus persistence probability of about 0.75 for an average viremia of 10 days and a maximum viremia of 154 days, corresponding to the probability of a viremia lasting longer than 70 days of less than 0.07. The available data for bluetongue thus seems to indicate that the long viremia in cattle could allow virus persistence. In this model, in contrast with most bluetongue virus serotypes, BTV8 can be vertically transmitted in a ruminant host at a relatively high rate: 0.1 to 0.3 of newborn calves with infected mothers are RT-PCR positive [5], [29]. The effective vertical transmissions must be less frequent insofar as positive RT-PCR does not necessarily signify infectivity. Furthermore, our model does not take into account that an infected cow only gives birth to a viraemic calf if the infection takes place during the second half of the pregnancy, the infection otherwise leading to an abortion or the birth of a susceptible calf [30]–[33]. However, the vertical transmission in a host appears as a very efficient means of persistence once the birth period corresponds to a favourable period for vectors since effective vertical transmission rates are 1% in humans or >1 ‰ in cattle. Chronic infection seems to be an effective virus persistence mechanism as theoretical persistence probabilities of between 0.5 and 1 can be obtained for short periods in the chronic stage (1 month) and low probabilities of passage to a chronic state and of a return to viremia (respectively 1.10*^−^* ^4^ and 1.10*^−^* ^4^ to 1.10*^−^* ^2^). However, the existence of such a phenomenon has only been demonstrated for the *Culex*/reptiles/Western equine encephalitis virus system [7]. No return to viremia could be demonstrated in birds for the viruses of Western equine encephalitis or St. Louis encephalitis [34], and for West Nile virus even if viral genome can be detected several weeks after infection [4].

The persistence of the virus in the vector’s overwintering stages reflects the adaptation of these arboviruses to the insects’ diapause mechanisms [3]. By combining this process with transovarian transmission, demonstrated for numerous arboviruses [4], [10], [35], [36], [37], the virus could persist many years, and all the more easily when the number of overwintering individuals and the probability of transmission are high. The range of theoretical values renders probable persistence by vertical transmission in the vector for the *Aedes*/dengue virus system, where vertical transmission is about 4% [37] and less certain for the *Culex*/West Nile virus system, where it is 7 to 8‰ [4]. This mechanism thus cannot always guarantee effective persistence, which will depend on the arbovirus/vector species considered [11].

Persistence being the same whatever the extrinsic incubation rate and the biting rate, a low continuous transmission does not seem to allow persistence (maximum values tested of 100 days and 1/100 days*^−^* ^1^); it is the possible survival of infected vectors that intervenes. It was demonstrated that in the *Culex*/West Nile virus system, females infected orally could conserve the virus at an undetectable level for 30 to 40 days at 10°C, and become infectious again as soon as the temperature increased [38]. However, overwintering *Culex* females do not usually feed blood and are therefore less likely to be infected, whereas bloodfed females are unlikely to enter diapause and survive winter, their average lifespan in laboratories being 20 days vs. 180 days for overwintering females [18]. The question remains open for viruses transmitted by *Culicoides* because it is possible to capture small quantities of these insects all winter long without being able to estimate their age [39].

Other persistence hypotheses exist that require more complex approaches to be taken into account. A multiplicity of hosts and/or of vectors may allow virus persistence past the unfavourable season [40], [41]. Two different mosquito genera, the *Culex* and *Aedes* ones, effectively are involved in the transmission of the Ross River and Rift Valley viruses, which may allow arboviruses to combine the persistence effects in each of these vectors [40]. The multiplicity of hosts could result in the adaptation of the virus to one species among the possible hosts which allows persistence, as in the case of cattle and bluetongue virus or zebra and horse sickness virus [42], [43]. The structure of the host and vector populations in time and space could influence virus persistence. The lifecycle of arthropods effectively is linked very closely to the climate [3], [44]. In our study, the function of seasonality only allows a single annual peak of vector abundance during the favourable period, although several peaks can be observed [45], [46]. This peak, which is sinusoidal and present over the entire favourable period, thus ensures the disappearance of the virus in the absence of any persistence mechanism. In addition, to not limit virus persistence within populations and to contradict its persistence or not during the unfavourable season, we chose to consider a non-limiting, large population of homogeneous hosts. A different approach by structuring the population in space (metapopulations, for example) would respond to other questions such as the impact of a rescue effect on virus persistence within numerous populations [47], as is possible to envision for West Nile virus, carried by migrating birds [48].

Our modelling approach allowed different arbovirus persistence mechanisms to be investigated. The survival of viruses within a host during the unfavourable season seems to be an effective strategy to adapt to seasonal variations and population dynamics of their vectors, either through extended viremia in a few individuals or through vertical transmission. The reactivation of chronic infections seems rare in arboviruses, although it is conventionally described for parasites, as in the case of malaria. Arboviruses with only a short presence in their hosts could persist through vertical transmission as seems likely for the dengue virus in humans. For viruses such as the West Nile virus, only new experimental investigations may allow overwintering modalities to be specified. Such investigations must aim to demonstrate a possible reactivation of chronic infection, which may be delicate given that a low occurrence can allow persistence, and explore abundances and survival of vectors during winter.

## References

[1] Tauber CA, Tauber MJ (1981) Insect seasonal cycles: genetics and evolution. AnnuRev Ecol Syst 12: 281–308.

[2] Dajoz R (2006) Mésoclimats et microclimats. Leur influence sur les êtres vivants. Précis d’écologie. Paris: Dunod. 57–83.

[3] Clements AN (1999) The biology of mosquitoes. Vol. 2: Sensory reception and behaviour. Wallingford: CABI Publishing. 740 p.

[4] Reisen WK, Fang Y, Lothrop HD, Martinez VM, Wilson J, et al (2006) Overwintering of West Nile Virus in Southern California. Journal of Medical Entomology 43(2): 344–355. 16619621 10.1603/0022-2585(2006)043[0344:oownvi]2.0.co;2

[5] Darpel KE, Batten CA, Veronesi E, Williamson S, Anderson P, et al (2009) Transplacental transmission of bluetongue virus 8 in cattle, UK. Emerg Infect Dis 15(12): 2025–2028. 19961692 10.3201/eid1512.090788 PMC3044536

[6] Maclachlan NJ, Drew CP, Darpel KE, Worwa G (2009) The Pathology and Pathogenesis of Bluetongue. Journal of Comparative Pathology 141(1): 1–16. 19476953 10.1016/j.jcpa.2009.04.003

[7] Thomas LA, Eklund CM (1962) Overwintering of Western Equine Encephalomyelitis Virus in Garter Snakes Experimentally Infected by Culex tarsalis. Proceedings of the Society for Experimental Biology and Medicine Society for Experimental Biology and Medicine (New York, NY) 109(2): 421–424. 10.3181/00379727-109-27225 13920821

[8] Bailey CL, Eldridge BF, Hayes DE, Watts DM, Tammariello RF, et al (1978) Isolation of St. Louis encephalitis virus from overwintering Culex pipiens mosquitoes. Science 199(4335): 1346–1349. 628843 10.1126/science.628843

[9] Rosen L, Tesh RB, Lien JC, Cross JH (1978) Transovarial transmission of Japanese encephalitis virus by mosquitoes. Science 199(4331): 909–911. 203035 10.1126/science.203035

[10] Rosen L (1987) Overwintering mechanisms of mosquito-borne arboviruses in temperate climates. The American journal of tropical medicine and hygiene 37(3): 69S–76S. 2891312 10.4269/ajtmh.1987.37.69s

[11] Adams B, Boots M (2010) How important is vertical transmission in mosquitoes for the persistence of dengue? Insights from a mathematical model. Epidemics 2(1): 1–10. 21352772 10.1016/j.epidem.2010.01.001

[12] Cruz-Pacheco G, Esteva L, Montaño-Hirose JA, Vargas C (2005) Modelling the dynamics of West Nile Virus. Bull Math Biol 67(6): 1157–1172. 16125762 10.1016/j.bulm.2004.11.008

[13] Charron MVP, Seegers H, Langlais M, Ezanno P (2011) Seasonal spread and control of Bluetongue in cattle. Journal of Theoretical Biology 291(0): 1–9. 21945148 10.1016/j.jtbi.2011.08.041

[14] Singer RS, MacLachlan NJ, Carpenter TE (2001) Maximal predicted duration of viremia in bluetongue virus-infected cattle. J Vet Diagn Invest 13(1): 43–49. 11243362 10.1177/104063870101300109

[15] Murray JO, Trainer DO (1970) Bluetongue Virus in North American Elk. Journal of Wildlife Diseases 6(3): 144–148. 4316480 10.7589/0090-3558-6.3.144

[16] Reeves WC, Hutson GA, Bellamy RE, Scrivani RP (1958) Chronic Latent Infections of Birds with Western Equine Encephalomyelitis Virus. Proceedings of the Society for Experimental Biology and Medicine Society for Experimental Biology and Medicine (New York, NY) 97(4): 733–736. 10.3181/00379727-97-23862 13554464

[17] Mitchell CJ (1988) Occurrence, biology, and physiology of diapause in overwintering mosquitoes. The arboviruses: epidemiology and ecology Volume I: 191–217.

[18] Mitchell CJ, Briegel H (1989) Inability of diapausing Culex-Pipiens (Diptera, Culicidae) to use blood for producing lipid reserves for overwinter survival. Journal of Medical Entomology 26(4): 318–326. 2769712 10.1093/jmedent/26.4.318

[19] Robich RM, Denlinger DL (2005) Diapause in the mosquito Culex pipiens evokes a metabolic switch from blood feeding to sugar gluttony. Proc Natl Acad Sci U S A 102(44): 15912–15917. 16247003 10.1073/pnas.0507958102 PMC1276097

[20] Jetten TH, Takken W (1994) Anophelism without malaria in Europe. A review of the ecology and distribution of the genus Anopheles in Europe. Wageningen Agricultural University Papers. 69 pp.

[21] Luedke AJ, Jones RH, Walton TE (1977) Overwintering mechanism for bluetongue virus: biological recovery of latent virus from a bovine by bites of Culicoides variipennis. Am J Trop Med Hyg 26(2): 313–325. 192095 10.4269/ajtmh.1977.26.313

[22] Gerry AC, Mullens BA, Maclachlan NJ, Mecham JO (2001) Seasonal Transmission of Bluetongue Virus by Culicoides sonorensis (Diptera: Ceratopogonidae) at a Southern California Dairy and Evaluation of Vectorial Capacity as a Predictor of Bluetongue Virus Transmission. Journal of Medical Entomology 38(2): 197–209. 11296823 10.1603/0022-2585-38.2.197

[23] Bonneau KR, DeMaula CD, Mullens BA, MacLachlan NJ (2002) Duration of viraemia infectious to Culicoides sonorensis in bluetongue virus-infected cattle and sheep. Veterinary Microbiology 88(2): 115–125. 12135632 10.1016/s0378-1135(02)00106-2

[24] Mullens BA, Gerry AC, Lysyk TJ, Schmidtmann ET (2004) Environmental effects on vector competence and virogenesis of bluetongue virus in Culicoides: interpreting laboratory data in a field context. In: MacLachlan NJ, Pearson JE, editors. Bluetongue, Pt 1, Proceedings. Teramo: Ist Zooprofilatttico Sperimentale Dell Abruzzo E Del Molise G Caporale. 160–166. 20419655

[25] Carpenter S, Lunt HL, Arav D, Venter GJ, Mellor PS (2006) Oral Susceptibility to Bluetongue virus of Culicoides (Diptera: Ceratopogonidae) from the United Kingdom. Journal of Medical Entomology 43(1): 73–78. 16506450 10.1093/jmedent/43.1.73

[26] Baylis M, O’Connell L, Mellor PS (2008) Rates of bluetongue virus transmission between Culicoides sonorensis and sheep. Medical and Veterinary Entomology 22(3): 228–237. 18816271 10.1111/j.1365-2915.2008.00732.x

[27] Gould EA, Higgs S (2009) Impact of climate change and other factors on emerging arbovirus diseases. Transactions of the Royal Society of Tropical Medicine and Hygiene 103(2): 109–121. 18799177 10.1016/j.trstmh.2008.07.025 PMC2915563

[28] Wilson A, Darpel K, Mellor PS (2008) Where does bluetongue virus sleep in the winter? PLoS Biol 6(8): 1612–1617. 10.1371/journal.pbio.0060210 PMC2525685 18752350

[29] Santman-Berends I, van Wuijckhuise L, Vellema P, van Rijn PA (2010) Vertical transmission of bluetongue virus serotype 8 virus in Dutch dairy herds in 2007. Veterinary Microbiology 141(1–2): 31–35. 19713058 10.1016/j.vetmic.2009.08.010

[30] De Clercq K, De Leeuw I, Verheyden B, Vandemeulebroucke E, Vanbinst T, et al (2008) Transplacental infection and apparently immunotolerance induced by a wild-type bluetongue virus serotype 8 natural infection. Transbound Emerg Dis 55(8): 352–359. 18673339 10.1111/j.1865-1682.2008.01044.x

[31] De Clercq K, Vandenbussche F, Vandemeulebroucke E, Vanbinst T, De Leeuw I, et al (2008) Transplacental bluetongue infection in cattle. Vet Rec 162(17): 564. 10.1136/vr.162.17.564 18441360

[32] Desmecht D, Bergh RV, Sartelet A, Leclerc M, Mignot C, et al (2008) Evidence for transplacental transmission of the current wild-type strain of bluetongue virus serotype 8 in cattle. Vet Rec 163(2): 50–52. 18621997 10.1136/vr.163.2.50

[33] Maclachlan NJ, Osburn BI (2008) Induced brain lesions in calves infected with bluetongue virus. Vet Rec 162(15): 490–491. 18408202 10.1136/vr.162.15.490-b

[34] Clements AN (2012) The biology of mosquitoes. Vol. 3: Transmission of viruses and interactions with bacteria: CABI Publishing. 571 p.

[35] Farajollahi A, Crans WJ, Bryant P, Wolf B, Burkhalter KL, et al (2005) Detection of West Nile Viral RNA from an Overwintering Pool of Culex pipens pipiens (Diptera: Culicidae) in New Jersey, 2003. Journal of Medical Entomology 42(3): 490–494. 15962803 10.1093/jmedent/42.3.490

[36] Nasci RS, Savage HM, White DJ, Miller JR, Cropp BC, et al (2001) West Nile virus in overwintering Culex mosquitoes, New York City, 2000. Emerg Infect Dis 7(4): 742–744. 11585542 10.3201/eid0704.010426 PMC2631767

[37] DeFoliart GR, Grimstad PR, Watts DM (1987) Advances in Mosquito-Borne Arbovirus/Vector Research. Annu Rev Entomol 32(1): 479–505. 2880554 10.1146/annurev.en.32.010187.002403

[38] Dohm DJ, Turell MJ (2001) Effect of incubation at overwintering temperatures on the replication of West Nile virus in New York Culex pipiens (Diptera : Culicidae). Journal of Medical Entomology 38(3): 462–464. 11372976 10.1603/0022-2585-38.3.462

[39] Losson B, Mignon B, Paternostre J, Madder M, De Deken R, et al (2007) Biting midges overwintering in Belgium. Vet Rec 160(13): 451–452. 10.1136/vr.160.13.451-b 17400907

[40] Glass K (2005) Ecological mechanisms that promote arbovirus survival: a mathematical model of Ross River virus transmission. Transactions of the Royal Society of Tropical Medicine and Hygiene 99(4): 252–260. 15708384 10.1016/j.trstmh.2004.08.004

[41] Lord CC (2009) The Effect of Multiple Vectors on Arbovirus Transmission. Isr J Ecol Evol 56(3–4): 371–392. 10.1560/IJEE.55.3-4.371 PMC3670802 23741205

[42] Mellor PS, Boorman J, Baylis M (2000) Culicoides biting midges: Their role as arbovirus vectors. Annu Rev Entomol 45: 307–340. 10761580 10.1146/annurev.ento.45.1.307

[43] Mellor PS, Hamblin C (2004) African horse sickness. Vet Res 35(4): 445–466. 15236676 10.1051/vetres:2004021

[44] Sanders CJ, Shortall CR, Gubbins S, Burgin L, Gloster J, et al (2011) Influence of season and meteorological parameters on flight activity of Culicoides biting midges. J Appl Ecol 48(6): 1355–1364.

[45] Balenghien T, Fouque F, Sabatier P, Bicout DJ (2006) Horse-, Bird-, and Human-Seeking Behavior and Seasonal Abundance of Mosquitoes in a West Nile Virus Focus of Southern France. Journal of Medical Entomology 43(5): 936–946. 17017231 10.1603/0022-2585(2006)43[936:hbahba]2.0.co;2

[46] Ponçon N, Toty C, L’Ambert G, Le Goff G, Brengues C, et al (2007) Population dynamics of pest mosquitoes and potential malaria and West Nile virus vectors in relation to climatic factors and human activities in the Camargue, France. Medical and Veterinary Entomology 21(4): 350–357. 18092973 10.1111/j.1365-2915.2007.00701.x

[47] Adams B, Kapan DD (2009) Man Bites Mosquito: Understanding the Contribution of Human Movement to Vector-Borne Disease Dynamics. PLoS ONE 4(8). 10.1371/journal.pone.0006763 PMC2727792 19707544

[48] Lopez G, Jimenez-Clavero A, Gomez Tejedor C, Soriguer R, Figuerola J (2008) Prevalence of West Nile Virus Neutralizing Antibodies in Spain Is Related to the Behavior of Migratory Birds. Vector-Borne and Zoonotic Diseases 8(5): 615–621. 18399777 10.1089/vbz.2007.0200

